# Variation in global codon usage bias among prokaryotic organisms is associated with their lifestyles

**DOI:** 10.1186/gb-2011-12-10-r109

**Published:** 2011-10-27

**Authors:** Maya Botzman, Hanah Margalit

**Affiliations:** 1 Department of Microbiology and Molecular Genetics, Institute of Medical Research Israel-Canada, Faculty of Medicine, The Hebrew University of Jerusalem, POB 12272, Jerusalem 91120, Israel

## Abstract

**Background:**

It is widely acknowledged that synonymous codons are used unevenly among genes in a genome. In organisms under translational selection, genes encoding highly expressed proteins are enriched with specific codons. This phenomenon, termed codon usage bias, is common to many organisms and has been recognized as influencing cellular fitness. This suggests that the global extent of codon usage bias of an organism might be associated with its phenotypic traits.

**Results:**

To test this hypothesis we used a simple measure for assessing the extent of codon bias of an organism, and applied it to hundreds of sequenced prokaryotes. Our analysis revealed a large variability in this measure: there are organisms showing very high degrees of codon usage bias and organisms exhibiting almost no differential use of synonymous codons among different genes. Remarkably, we found that the extent of codon usage bias corresponds to the lifestyle of the organism. Especially, organisms able to live in a wide range of habitats exhibit high extents of codon usage bias, consistent with their need to adapt efficiently to different environments. Pathogenic prokaryotes also demonstrate higher extents of codon usage bias than non-pathogenic prokaryotes, in accord with the multiple environments that many pathogens occupy. Our results show that the previously observed correlation between growth rate and metabolic variability is attributed to their individual associations with codon usage bias.

**Conclusions:**

Our results suggest that the extent of codon usage bias of an organism plays a role in the adaptation of prokaryotes to their environments.

## Background

The genetic code is composed of triplets of four nucleotide types for 20 amino acids. This redundancy implies the use of synonymous codons - different codons encoding the same amino acid. Synonymous codons may differ in their frequency of occurrence among different genes within an organism, a phenomenon known as 'codon usage bias' [1]. It was demonstrated that many bacteria and yeast undergo translational selection, with highly expressed genes preferentially using codons assumed to be translated faster and/or more accurately by the ribosome [2,3]. Previous works suggested that these codons are the ones matching abundant tRNAs, which are organism-specific [3-7]. Other works demonstrated additional factors affecting the frequencies of the synonymous codons in an organism, such as the genome GC content [8,9]. Thus, the preferred codons per amino acid may vary between different organisms, based on their tRNA repertoire and other factors.

While it was claimed that most prokaryotes undergo translational selection [10], it is conceivable that various organisms may differ in the extent of codon usage bias across their genes and in the forces determining it. For several organisms, such as *Escherichia coli *and *Saccharomyces cerevisiae*, a positive correlation between codon bias of genes and their protein levels was demonstrated (for example, [11,12]), suggesting that in those organisms translational selection is predominant. Other organisms, such as *Helicobacter pylori *[13], show almost no differential use of synonymous codons among different genes. While in the former organisms a mixture of mutation bias and natural selection underlies codon usage bias, in the latter organisms codon usage bias is mostly explained by mutation and very weak, if any, translational selection [14]. It was suggested by Andersson and Kurland [15] and recently substantiated by Kudla *et al*. [16] that selection towards highly adapted codons in highly expressed proteins has a global effect on the cell. By this convention, high expression of certain genes is mainly achieved by various mechanisms, such as regulation of transcription and/or translation initiation, and the use of well adapted codons in these genes guarantees efficient recycling of the ribosomes, resulting in an increase in cellular fitness. This might be reflected in a relatively enhanced translation of the whole proteome. It is thus intriguing to examine the association between the extent of codon usage bias of various organisms and their phenotypic traits towards understanding the environmental conditions where high extent of codon usage bias is advantageous. Of note, difference in the usage of synonymous codons that is uniform across all genes in a genome and probably arises from non-selective processes is not addressed here, but the different usage of codons among genes in a genome.

We regard an organism as 'biased' or 'unbiased' in association with its codon usage if the distribution of synonymous codons in its highly expressed genes differs from that in other genes in the genome. Several measures were proposed to estimate the extent of codon usage bias at an organism scale, enabling the classification of an organism as biased or unbiased [14,17-19]. Here we present such a measure based on the Codon Adaptation Index (CAI) [20] of individual genes. The CAI of a gene ranges between 0 and 1, with higher values indicating the use of more preferred codons. The extent of preference of each codon is determined by its frequency among the codons in genes encoding ribosomal proteins, using the latter as proxy for highly expressed genes. An organism-scale measure is obtained by computing CAI_ave_, the average of the CAI values of all genes in a genome [17]. Since per definition highly expressed genes are assigned high CAI values, low or high CAI_ave _values indicate whether there is a difference in codon usage between highly expressed genes and the rest of the genes in a genome. A low CAI_ave _of an organism implies that there are many genes with low CAI values, and therefore preferred codons are assigned only to a small group of highly expressed genes. Accordingly, low CAI_ave _values are indicative of biased organisms. A high CAI_ave _of an organism implies that the CAI values of most genes are similar to those of genes encoding the ribosomal proteins, and therefore there is no differential use of synonymous codons among the genes encoded in that organism. Such organisms are unbiased in regard to their codon usage. Another measure for the extent of codon usage bias of an organism is based on another measure of gene codon usage bias, the Nc (the effective number of codons) [21]. This gene measure ranges between 20 and 61, with lower values indicating the use of less codon types per amino acid along a protein-coding gene, which most often are the more preferred codons. To evaluate the extent of codon usage bias of an organism, a measure based on the difference between the average Nc values of the ribosomal genes and the average Nc values of the rest of the genes in the genome, Nc_diff_, is computed [17]. Organisms with high values of Nc_diff _exhibit large extents of codon usage bias, and vice versa. The two genomic measures, CAI_ave _and Nc_diff_, are highly correlated (Figure S1 in Additional file 1; Pearson r = -0.91, *P *≈ 0, *n *= 1,169).

Here we used these measures to characterize the extent of codon usage bias in 773 prokaryotic species representing 1,169 sequenced genomes. We demonstrate a wide range in the extent of codon usage bias among the various organisms, and trace the possible sources of this variation. Our results indicate that similarity in the extents of codon usage bias of different organisms reflects a similar ecological strategy they share.

## Results

### Organisms differ in their extent of codon usage bias

We analyzed the extent of codon usage bias in 773 organisms: 699 bacteria and 74 archaea, representing 1,169 genomes (Materials and methods). Since the results for CAI_ave _and NC_diff _are highly correlated, we present here the results for the first measure and in Additional file 1 the results for the second measure (Figures S2, S4, S5 and S6 in Additional file 1).

**Figure 1:**
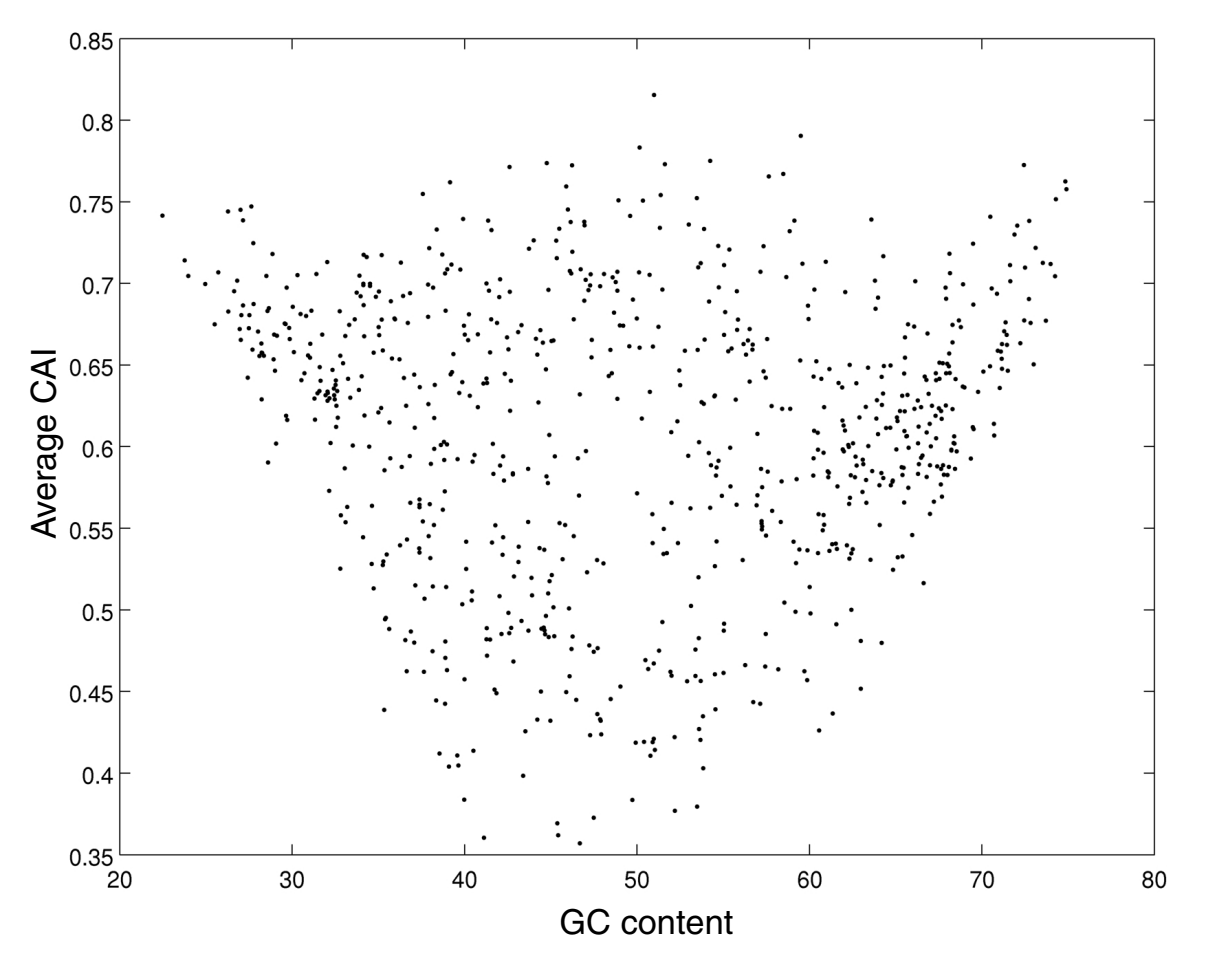
**Prokaryotes showing extreme GC content have relatively high CAI_ave _values spanning a narrower range**. Scatter plot of GC content (x-axis) and CAI_ave _(y-axis) of 773 organisms. Here and in all other figures CAI_ave _is indicated as 'Average CAI'. CAI: Codon Adaptation Index; GC: genome content.

We first examined the association between the genomic values of CAI_ave _and GC content (Figure 1), and observed that prokaryotes with extreme GC contents exhibit a narrower range of CAI_ave _values than the other prokaryotes. Such organisms cannot achieve low CAI_ave _values because their extreme GC content defines a limited repertoire of codons that are shared by both ribosomal genes and the rest of the genes, thus resulting in unbiased genomes (high CAI_ave_). To avoid bias in our conclusions due to the inclusion of organisms with extreme GC content, these organisms were excluded from further analyses. The next analyses were carried out for prokaryotes with GC contents between 35% and 65%, a total of 518 organisms.

**Figure 2:**
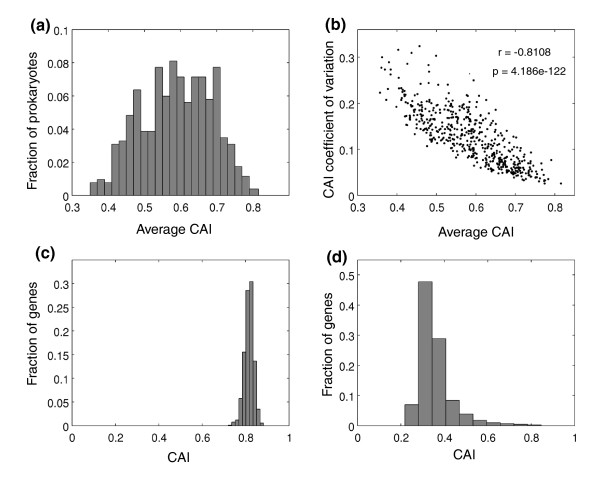
**Wide variation in the extents of codon usage bias among various organisms**. **(a) **Distribution of CAI_ave _values among 518 prokaryotes with GC content between 35% and 65%. CAI_ave _is indicated as 'Average CAI'. **(b) **Negative correlation between organisms' CAI_ave _values and their coefficients of variation. **(c) **Distribution of CAI values among the genes of *Syntrophothermus lipocalidus *(high CAI_ave_, indicating low extent of codon usage bias). **(d) **Distribution of CAI values among the genes of *Vibrio vulnificus *(low CAI_ave_, indicating high extent of codon usage bias). CAI: Codon Adaptation Index; GC: genome content.

As shown in Figure 2a, there is a wide distribution of CAI_ave _values across the genomes, ranging between 0.35 (most biased organisms) and 0.82 (least biased organisms), with an average value of 0.59, median of 0.59 and standard deviation of 0.097. Another informative genomic measure is the standard deviation of CAI values of genes in a genome, which indicates the breadth of values used in calculating the genomic CAI_ave _measures. To get an impression of this property across genomes we computed for each genome the coefficient of variation of CAI values of genes (standard deviation divided CAI_ave_)_. _As expected, we find a strong inverse correlation between the CAI_ave _values and their coefficients of variation (Figure 2b; Pearson r = -0.81, *P *= 4.186E-122, *n *= 518), indicating that the distribution of CAI values of genes in biased organisms (low CAI_ave_) is broader than that of CAI values of genes in unbiased organisms.

To substantiate this result we investigated in each genome the difference in the usage of each codon between highly expressed genes and the rest of the genes encoded in the genome. For each organism we calculated the frequency of each codon out of the total codons encoding the corresponding amino acid, once in the ribosomal genes and once in the rest of the genes. We then calculated the average difference between codon frequencies in the ribosomal genes and in the rest of the genes. Comparison of these average differences and CAI_ave _values across genomes revealed a strong negative correlation (Pearson r = -0.8593, *P *= 2.2639E-152, *n *= 518). This result reinforces our view that organisms with low CAI_ave _are under stronger translational selection, resulting in differences between the codons in the highly expressed genes and the codons in the rest of the genes, while in organisms with high CAI_ave _there is almost no differential use of codons between highly and weakly expressed genes. The latter unbiased organisms can be of two types: either the synonymous codons of both ribosomal and other genes are determined based on the GC content of the genome, or most genes use the preferred codons (that fit the abundant tRNAs or follow other rules that determine codon preference). To this end we looked at the association between CAI_ave _and the average Nc of a genome in all 1,169 organisms. Low average Nc values imply that all genes in the genome use a specific set of codons and high average Nc values represent genomes where all genes use a mixture of codons. Indeed, we find among genomes with high CAI_ave _these two types of trends (Figure S3a in Additional file 1). As to the biased genomes (with CAI_ave _below the average of 0.59), most of them use relatively many codons across their genes (average Nc values above 40), but apparently they use specific codons in their highly expressed genes, represented by the ribosomal genes. In general, it seems that the average values of Nc are strongly determined by the GC content of the genome (Figure S3b in Additional file 1). Especially, as we noted above, in genomes with extreme GC content, where the possibilities of codon variation are severely restricted by the specific nucleotide repertoire, the average Nc values are low.

Organisms at the right tail of the CAI_ave _distribution are the least biased. The prokaryote with the smallest extent of codon usage bias (highest CAI_ave _value, 0.82) is *Syntrophothermus lipocalidus*, a thermophilic, syntrophic, fatty-acid-oxidizing anaerobe that belongs to the Clostridia class [22] (Figure 2c). Other prokaryotes with almost as high CAI_ave _values are quite diverse: *Geobacter metallireducens *(CAI_ave _value of 0.79) is an anaerobic bacterium that uses iron oxides as the electron acceptor in the oxidation of organic compounds to carbon dioxide [23], *Nitrosococcus watsoni *(CAI_ave _value of 0.78) is an aerobic marine bacterium, and *Coxiella burnetti *(CAI_ave _value of 0.78) is a facultative, intracellular pathogenic bacterium that causes the Q fever.

**Figure 3:**
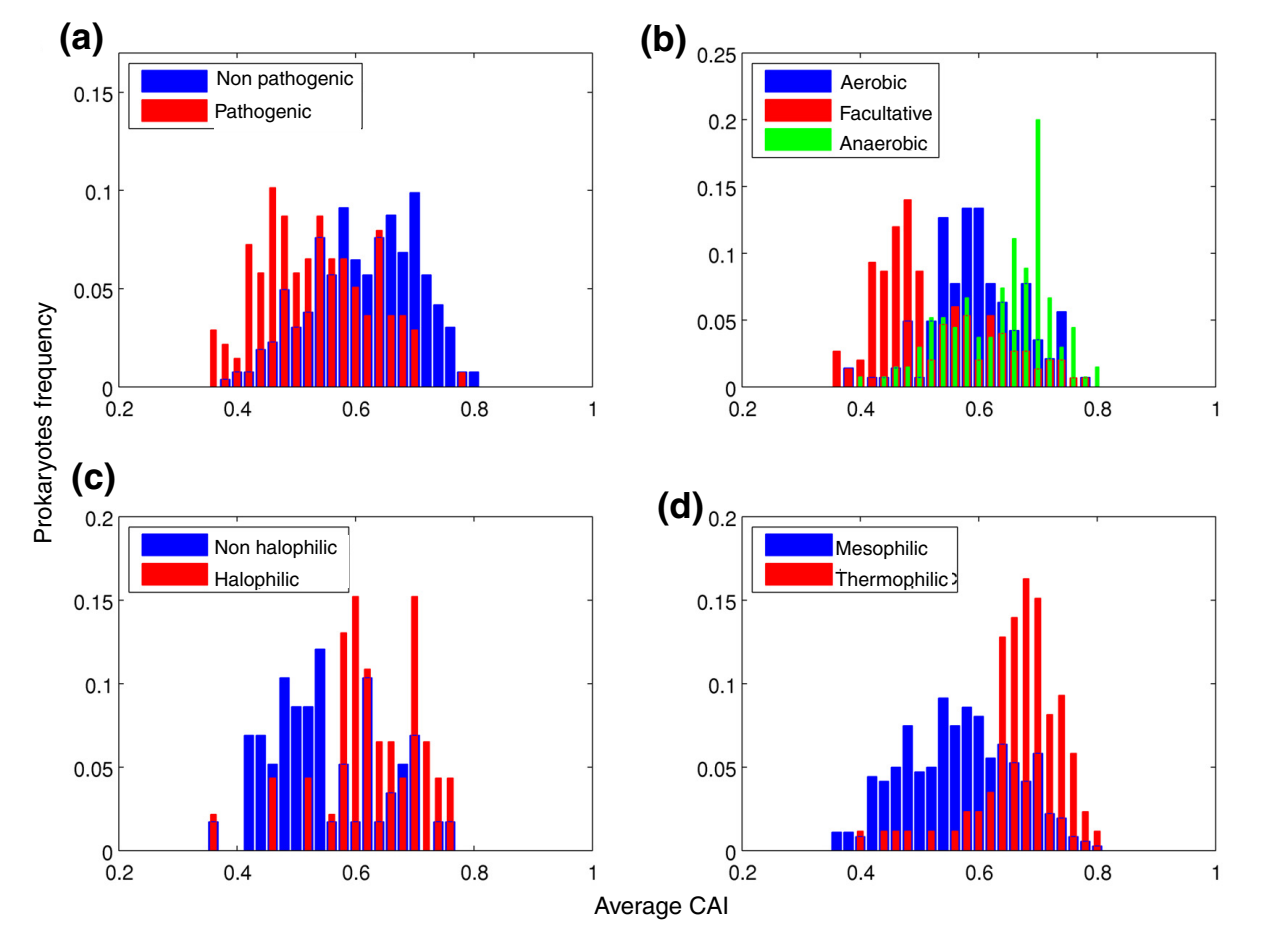
**Prokaryotes exhibiting different environmental characteristics show different extents of codon usage bias**. The 518 prokaryotes were classified according to various properties, and the distributions of CAI_ave _values of the different classes were compared. **(a) **Pathogenic prokaryotes show larger extents of codon usage bias than non-pathogenic prokaryotes. CAI_ave _is indicated as 'Average CAI'. **(b) **Facultative prokaryotes show the largest extent of codon usage bias and anaerobic prokaryotes show the smallest extent. **(c) **Prokaryotes that live in different salinity environments show a statistically significant difference in CAI_ave _values. **(d) **Thermophilic and hyperthermophilic prokaryotes show a smaller extent of codon usage bias than mesophilic prokaryotes. CAI: Codon Adaptation Index; GC: genome content.

Organisms at the left tail of the distribution are the most biased. The prokaryote that exhibited the greatest extent of codon usage bias (lowest CAI_ave _value, 0.35) is *Vibrio vulnificus*, a human pathogen of the Gammaproteobacteria class (Figure 2d). Interestingly, other organisms that have such large extents of codon usage bias are also pathogenic: *Streptococcus suis *is a swine pathogen, *Vibrio cholera *causes cholera in humans and *Corynebacterium diphtheria *causes diphtheria, an upper respiratory tract illness (CAI_ave _values of 0.36, 0.37, and 0.38, respectively). This finding led us to examine the distribution of CAI_ave _values among pathogenic versus non-pathogenic prokaryotes (Figure 3a). As shown in Figure 3a, the distribution among pathogenic bacteria is biased to the left compared to non-pathogenic bacteria, with pathogenic prokaryotes having statistically significant lower CAI_ave _values (*P *= 1.26E-15 by Mann-Whitney test), indicating they are more biased.

### Biased organisms can be classified by their phenotypic traits

We annotated each genome with its CAI_ave_, NC_diff _and several phenotypic traits, such as its oxygen requirement and range of growth temperatures (Additional file 2). This annotation system (Table 1) has enabled us to compare the distributions of the CAI_ave _values of different groups of organisms, tagged according to a particular phenotypic trait.

**Table 1:** Classification of organisms by phenotypic traits

Environmental property	Number of prokaryotes
**Pathogenicity**	
Pathogenic	138
Non-pathogenic	263
Total	401
	
**Oxygen requirement**	
Aerobic	142
Anaerobic	135
Facultative	150
Microaerophilic	12
Total	439
	
**Salinity**	
Extreme halophilic	7
Mesophilic	19
Moderate halophilic	20
Non-halophilic	58
Total	104
	
**Temperature range**	
Hyperthermophilic	35
Mesophilic	361
Psychrophilic	12
Thermophilic	51
Total	459
	
**Habitat**	
Multiple	127
Specialized	81
Total	208

**Figure 4:**
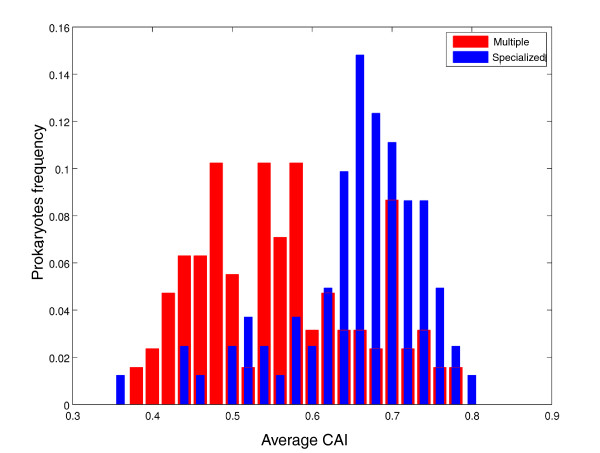
**The ability of an organism to live in multiple habitats is the feature most correlated with the extent of codon usage bias**. The 518 prokaryotes were divided into two groups - organisms who each live in a specialized environment and organisms who each live in multiple habitats - and the CAI_ave _values of the different groups were compared. CAI_ave _is indicated as 'Average CAI'. CAI: Codon Adaptation Index; GC: genome content.

This analysis (Figure 3) indicated that groups of prokaryotes classified by their oxygen requirement differ statistically significantly in the distributions of CAI_ave _values (*P *= 1.99E-23 by Kruskal-Wallis test). Facultative organisms exhibited the largest extent of codon usage bias and anaerobic organisms showed the smallest values (*P *= 2.92E-12, Mann-Whitney test between facultative and aerobic; *P *= ~0 between facultative and anaerobic; *P *= 3.14E-6 between aerobic and anaerobic). Examining groups of prokaryotes that live in environments that differ in their salinity levels demonstrated statistically significant differences among them (*P *= 2.13E-5 by Mann-Whitney test). Organisms that live in different temperature ranges showed statistically significant differences in their CAI_ave _values (*P *= 5.52E-21 by Mann-Whitney test): thermophiles demonstrated statistically significantly higher CAI_ave _values than mesophiles. Intriguingly, we found that organisms living in multiple habitats have statistically significantly lower CAI_ave _values than organisms living in specialized habitats (Figure 4; *P *= 6.3E-10 by Mann-Whitney test). This result is consistent with the results presented above for the other phenotypic traits and generalizes them. Pathogenic bacteria often live in multiple environments outside and within their host, and facultative organisms live in environments with and without oxygen. On the other hand, thermophiles (found above to be less biased than mesophiles) are usually restricted to a specific environment with a specific temperature. The consistency between these results is also implied by the interdependence between these different phenotypic traits (as shown by χ^2 ^test; Table S2 in Additional file 1).

### Phenotypic traits rather than phylogenetic relatedness underlie the similarities in codon usage bias between organisms

**Figure 5:**
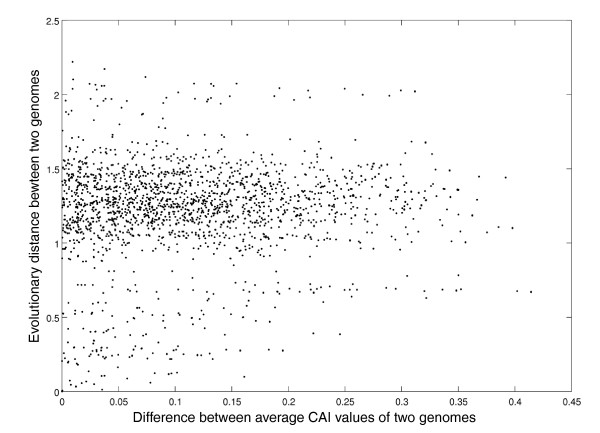
**No correlation between pairwise evolutionary distances across the phylogenetic tree and difference in CAI_ave _values of pairs of organisms**. Scatter plot of the difference in CAI_ave _values between each pair of two organisms in our data (x-axis) and their phylogenetic distances (y-axis). CAI_ave _is indicated as 'Average CAI'. The pairwise distances across the phylogenetic tree were based on the tree generated in [38] and computed as the path length between two organisms through the most recent common ancestor. CAI, Codon Adaptation Index.

It is possible that biased organisms are evolutionarily related and their similar values of CAI_ave _stem from their phylogenetic relatedness. To test this, we computed the correlation coefficient between the phylogenetic distance (Materials and methods) and difference in CAI_ave _values of pairs of organisms. The pairwise measures were computed for pairs of prokaryotes and pairs of archaea, and the correlation analysis was carried out for all pairs together (Figure 5). No correlation was found between the phylogenetic distances and the differences in CAI_ave _(Pearson r = 0.078), implying no noticeable influence of the phylogeny on the extent of codon usage bias. As shown in Figure 5, organisms with very small differences in their CAI_ave _values can be distantly separated on the evolutionary tree. Of note, organisms that are extremely close on the phylogenetic tree do not exhibit differences in CAI_ave _that are larger than 0.15. Thus, in these cases the similarity in CAI_ave _values may stem from the close phylogenetic relatedness. However, beyond a certain (low) threshold, there is no dependence between the phylogenetic distance and extent of codon usage bias. These results strengthen our previous conclusion that the extent of codon usage bias is associated with the phenotypic traits of the organism.

### The empirical association between growth rate and metabolic variability is attributed to their individual associations with codon usage bias

Previous studies showed that there is an association between codon bias and growth rate [17,24] and between growth rate and metabolic variability [25]. To verify that our result is not indirectly inferred from these two associations, we computed the correlation coefficient between pairs of properties (CAI_ave_, growth rate, and type of habitat (multiple or specific)). This analysis included 82 organisms for which we had information on their growth rate and habitat type. We repeated this analysis twice. Once we simply computed the correlation coefficient of two variables, and in the second analysis we performed partial correlation, controlling for the third variable (Table 2a). This analysis demonstrates that our conclusion about an association between CAI_ave _and the type of habitat is independent of the correlations with the growth rate, as the two correlation coefficients obtained in the two computations, with and without taking into account the growth rate, were very similar (r = 0.46, *P *= 1.25E-5 and r = 0.43, *P *= 6.9E-5, respectively). Intriguingly, the correlation between growth rate and habitat type was shown to be highly dependent on CAI_ave_. While the correlation between these two variables was found to be approximately 0.2 and nearly statistically significant (*P *= 0.07), the partial correlation, controlling for CAI_ave_, dropped substantially to 0.04 (*P *= 0.6). Our results suggest that the empirical association observed between growth rate and metabolic variability can be attributed to their individual associations with codon usage bias. Of note, the correlation between CAI_ave _and habitat type was the highest obtained and it is highly statistically significant. To verify that our conclusions are not affected by inclusion of closely related organisms, which might introduce redundancy in the data, we repeated the analysis with a subset of the 82 organisms, including 24 organisms that are phylogenetically remote (Materials and methods; Table 2, Analysis B). Using this dataset, the correlations between growth rate and both habitat type and codon bias were not statistically significant. The correlation and partial correlation between CAI_ave _and habitat type were consistent with the results for the whole dataset.

**Table 2:** Correlations and partial correlations between growth rate, habitat type and global codon usage bias

	Growth rate and habitat type		Growth rate and codon usage bias		Habitat type and codon usage bias	
	**r**	***P***	**r**	***P***	**r**	***P***
**Analysis A**						
Correlation	0.1995	0.0723	0.3451	0.0015	0.4619	1.2538E-5
Partial correlation	0.0482^a^	0.6693^a^	0.291^b^	0.0084^b^	0.4274^c^	6.919E-5^c^
**Analysis B**						
Correlation	0.0368	0.8645	0.2971	0.1586	0.4692	0.0207
Partial correlation	NA	NA	NA	NA	0.4802^c^	0.0204^c^

Analysis A was based on 82 organisms; analysis B was based on a subset of 24 organisms, which are phylogentically remote from each other. Pearson correlation coefficients were computed. ^a^Controlling for codon usage bias; ^b^controlling for habitat type; ^c^controlling for growth rate. NA, when the Pearson correlation coefficient was not statistically significant, partial correlation was not computed.

## Discussion

It is widely acknowledged that synonymous codons are used unevenly among genes in a genome, with genes encoding highly expressed proteins being enriched with specific codons. It is still under debate whether the biased use of certain codons in highly expressed genes is one of the causes or the result of the high expression level. On the one hand, it was shown that the use of certain codons affects directly the speed of translation [26] and its accuracy [27], and codon optimization is even used to elevate the levels of proteins expressed outside their original context [28,29]. On the other hand, Kudla *et al*. [16] showed that the variation in the levels of proteins translated from synthetic green fluorescent protein constructs, varying only at synonymous sites, was not correlated with the codon usage. They found that high expression was not associated with specific codons but with avoidance of secondary structure at the translation initiation site. This supports the proposition [15] that selection for well adapted codons in highly expressed genes does not affect directly the level of individual proteins, but provides a global benefit to the cell, as it assures efficient recycling of the ribosomes, which leads to an increase in cellular fitness. It should be noted that avoidance of secondary structure at the 5' end of the mRNA is only one mechanistic strategy that may lead to high levels of gene expression [30], and other mechanisms could underlie high levels of expression as well. These include high transcription level from strong promoters, high stability of the mRNA and/or efficient translation initiation by optimal Shine-Dalgarno sequences. Thus, different highly expressed genes might use well adopted codons to improve cellular fitness, independent of the molecular mechanism underlying their high expression. The premise by which translation selection for preferred codons in highly expressed genes has a global effect motivated us to investigate its association with the phenotypic traits of a wide range of organisms. It should be noted that our conclusions are not affected by whether or not the suggested global effect is accompanied by local effects on the translation efficiency.

There have been various attempts to explain what makes certain codons preferred over others in highly expressed genes, from correspondence to abundant tRNAs to physical considerations regarding the optimal stability of codon-anticodon interaction (summarized in [17]). Here we have not dealt with these various possible types of preferred codons but regarded them as the codons used by highly expressed genes in a genome, based on the premise that selection favored the most adapted codons in highly expressed genes. Hence, our analysis was based on measures that compare the codon usage of all genes to the codon usage of highly expressed genes, represented by the set of ribosomal genes in each genome (CAI_ave _and Nc_diff_). Such a comparative measure should provide us information on the selection forces that act on individual genes and on the whole genome. Indeed, we find a wide variation in these measures across genomes (Figure 2a; Figure S2 in Additional file 1), where some organisms are highly biased and others show only very slight, if any, difference between the codon usage in ribosomal genes and other genes. It is possible that the lack of selection implied for some of the unbiased genomes actually reflects their small population size [31], but in the absence of a reliable measure of effective population size in bacteria, we are unable to assess this further. In biased genomes selection acts only on genes that are highly expressed, to assure the overall translation mechanism to operate efficiently. Thus, our analysis is in line with previous observations [17] that there are organisms where translational selection is operational (biased genomes) and others where it is not (unbiased). In our study we used the set of ribosomal genes as a representative set of highly expressed genes. When data of gene expression in many prokaryotes become available it should be possible to extend our study by using sets of highly expressed genes based on their measured expression levels. It would be possible then to divide the organisms into those with high and low variation in gene expression, and to examine how the level of variation in gene expression is reflected in CAI_ave _values.

We found that pathogenic prokaryotes have statistically significantly lower CAI_ave _than non-pathogenic prokaryotes, but with a substantial overlap between the histograms of those two groups. This result might be surprising in view of previous studies that found that pathogenic lifestyle is linked to relaxation of selection [32-34], which should be linked to reduced codon bias. It is possible to reconcile the discrepancy by dividing the pathogenic and the non-pathogenic prokaryotes into two subtypes: some are capable of living in multiple environments and some stay mainly host-associated. When we compared pathogenic and non-pathogenic groups of host-associated prokaryotes there was no statistically significant difference in their CAI_ave _values (*P *= 0.06 by Mann-Whitney test), but when we compared pathogenic and non-pathogenic groups of prokaryotes living in multiple habitats, the pathogenic prokaryotes showed statistically significantly lower CAI_ave _values (*P *= 5.624E-7 by Mann-Whitney test). Therefore, it seems that pathogenic host-associated microbes like *C. burnetti*, which is an intracellular pathogen with an extremely high CAI_ave _value of 0.78, are not exposed to strong selection, while other pathogens that are able to live in multiple habitats are still under stronger selection than non-pathogenic prokaryotes living in multiple habitats when it comes to selection on codon usage.

Previous studies discussed the correspondence between ecology preferences and codon adaptation [35,36]. Our results suggest that organisms may adjust to metabolic variability by maintaining a high extent of codon usage bias (reflected by their low CAI_ave _values). Previous studies analyzed the association between codon adaptation and growth rate and between growth rate and metabolic variability [14,17,24,25]. One study showed that most bacterial organisms choose one of two alternative ecological strategies: living in multiple habitats with a large extent of co-habitation or living in a specialized niche in which the co-habitation is limited. It was shown that growth rate is statistically significantly correlated with metabolic variability encountered by an organism, suggesting a universal principle by which metabolic flexibility is associated with a need to grow fast, perhaps because of the greater extent of competition [25]. Independently, Rocha demonstrated an association between bacteria with large extents of codon usage bias and fast growth, and also an association with the number of tRNA genes [17,24]. It was demonstrated that fast growing bacteria have more tRNA genes of fewer types, and suggested that the translation in those organisms depends on fast tRNA diffusion to the ribosome. That study proposed that co-evolution of the tRNA pool and the codon usage bias allows more efficient translation of highly expressed genes, and that the codon usage bias in highly expressed genes relative to the rest of the genome is predicted to be under stronger selection in fast growing organisms. Our results tie these two results together and suggest that translational selection towards most adapted codons in highly expressed genes is operational in organisms that live in variable environments, enabling them to efficiently address the metabolic variability and the competition.

## Conclusions

Codon usage bias in highly expressed genes was suggested to have a global effect on the cell, increasing cellular fitness. Here we perform the first large-scale study that examines the relationship between codon usage bias and the phenotypic traits of prokaryotic organisms. Our analysis revealed a large variation in the global extent of codon bias among prokaryotic genomes, which is associated with their lifestyles. Especially, we discovered that organisms living in multiple habitats, including facultative organisms, mesophiles and pathogenic bacteria, exhibit high extents of codon usage bias, consistent with their need to adapt efficiently to different environments.

## Materials and methods

### Data organization

We retrieved 1,169 genome sequences from the NCBI Entrez Genome Project database [37]. For species that had sequenced genomes for more than one subspecies, we maintained one representative subspecies, which was chosen randomly. This resulted in a data set of 773 prokaryotes. From this list, only prokaryotes with GC content larger than 35% and smaller than 65% were included for further analyses, resulting in a data set of 518 prokaryotes.

### Computation of codon bias measures at an organism scale

Genome CAI_ave_: for each organism we computed the average of CAI values over all genes in the genome [17]. The CAI of a gene was computed as described in [20].

NC_diff_: for each organism we calculated the Nc value for each gene in the genome, as described in [21]. We next computed the average Nc value of the genes encoding ribosomal proteins (*Nc *(*rib*)) and the average Nc value of the rest of the genome (*Nc *(*all*)) [17]. NC_diff _was obtained by:

$$\frac{Nc\left(all\right)-Nc\left(rib\right)}{Nc\left(all\right)}$$

### Comparison between groups of organisms

The prokaryotes were classified according to their environmental characteristics (oxygen requirement, salinity, temperature range and habitat) and also whether they are pathogenic, based on the documentation in the NCBI Entrez Genome Project database (detailed in Additional file 2). The number of organisms annotated with each property is detailed in Table 1. Comparisons between the value distributions of organism measures (CAI_ave _or NC_diff_) were performed by Mann-Whitney or Kruskal-Wallis tests.

### Phylogenetic distances between organisms

The pairwise distances across the phylogenetic tree were based on the tree generated in [38] and computed as the path length between two organisms through the most recent common ancestor. We used 2,016 possible pairs of 64 bacteria and 78 possible pairs of 13 archaea in this analysis.

### Correlations and partial correlations between growth rate, habitat type and codon usage bias

This analysis included 82 prokaryotes, for which we had information for both their growth rates and habitat types. The growth rates were obtained from [24]. The prokaryotes' environmental annotations were obtained from the NCBI Entrez Genome Project database (multiple habitat-living organisms were annotated as 0, and specialized organisms were annotated as 1). The extents of codon usage bias were represented as CAI_ave _values. We repeated the analysis using a subset of 24 organisms, which are all phylogentically remote from each other, based on the phylgenetic tree used above [38].

## Abbreviations

CAI: Codon Adaptation Index.

## Competing interests

The authors declare that they have no competing interests.

## Authors' contributions

MB carried out all analyses, conceived the work, analyzed the data and wrote the paper. HM conceived the work, analyzed the data and wrote the paper. All authors have read and approved the manuscript for publication.

## Supplementary Material

Additional file 1 **Additional figures and tables**. Click here for file

Additional file 2 **Table S1 - features and characteristics of the prokaryotes included in this study**. This file contains data and annotations of the organisms included in the analysis (nam, tax ID, CAI_ave_, median CAI, coefficient of variation (of CAI), Nc_diff_, average Nc, environmental properties, if it is the representative subspecies of the species, super kingdom, GC content). Click here for file
