# Supplementary material for: Variation in global codon usage bias among prokaryotic organisms is associated with their lifestyles
Source: Genome Biol. 2011 Oct 27;12(10):R109. doi: 10.1186/gb-2011-12-10-r109 (PMC3333779; doi:10.1186/gb-2011-12-10-r109)
Supplement: Additional file 1 — Additional figures and tables. [file gb-2011-12-10-r109-S1.PDF]

Variation in global codon usage bias among prokaryotic organisms is associated with their lifestyles

Maya Botzman and Hanah Margalit

Additional File 1: Additional figures and tables

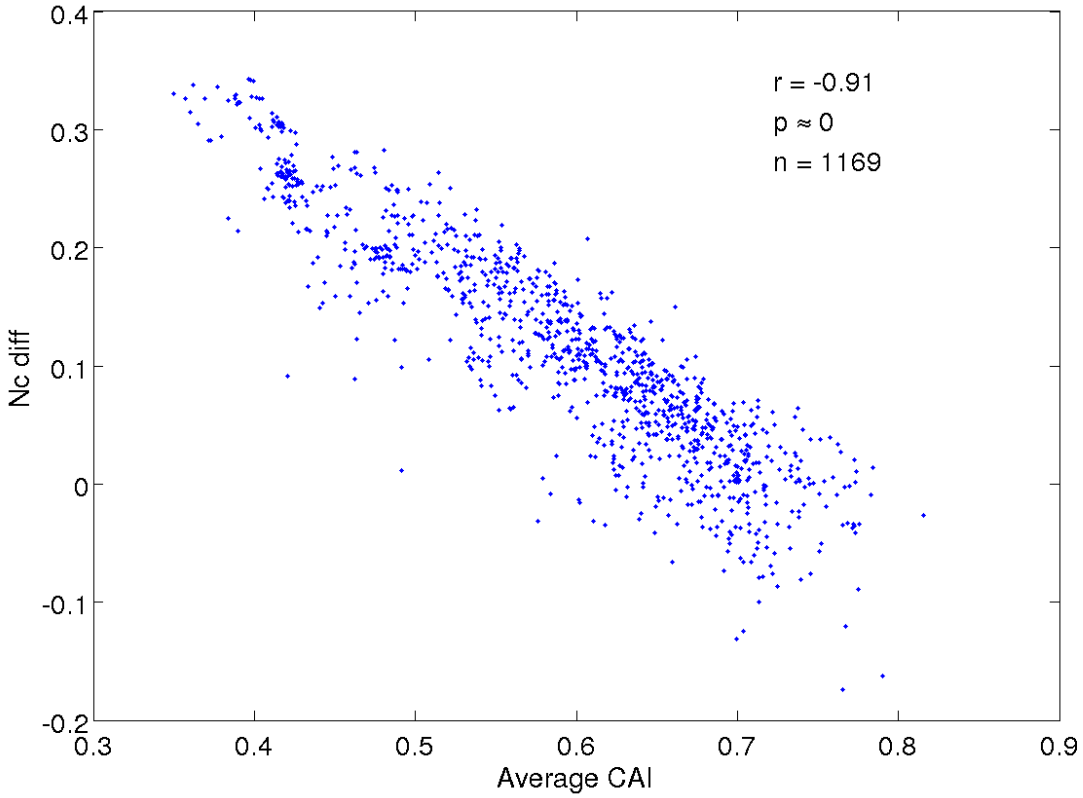

Figure S1. CAI_{ave} and Nc_{diff} are highly correlated. For each prokaryote CAI_{ave} value was plotted against Nc_{diff} value. Nc_{diff} is computed as the difference between the average of Nc value of the ribosomal genes (Nc(rib)) and the average of Nc values of the rest of the genes (Nc(all)) normalized by Nc(all). A high negative correlation is observed, indicating that the two measures can be used interchangeably to evaluate the extent of codon usage bias of an organism. CAI_{ave} is indicated as 'Average CAI'.

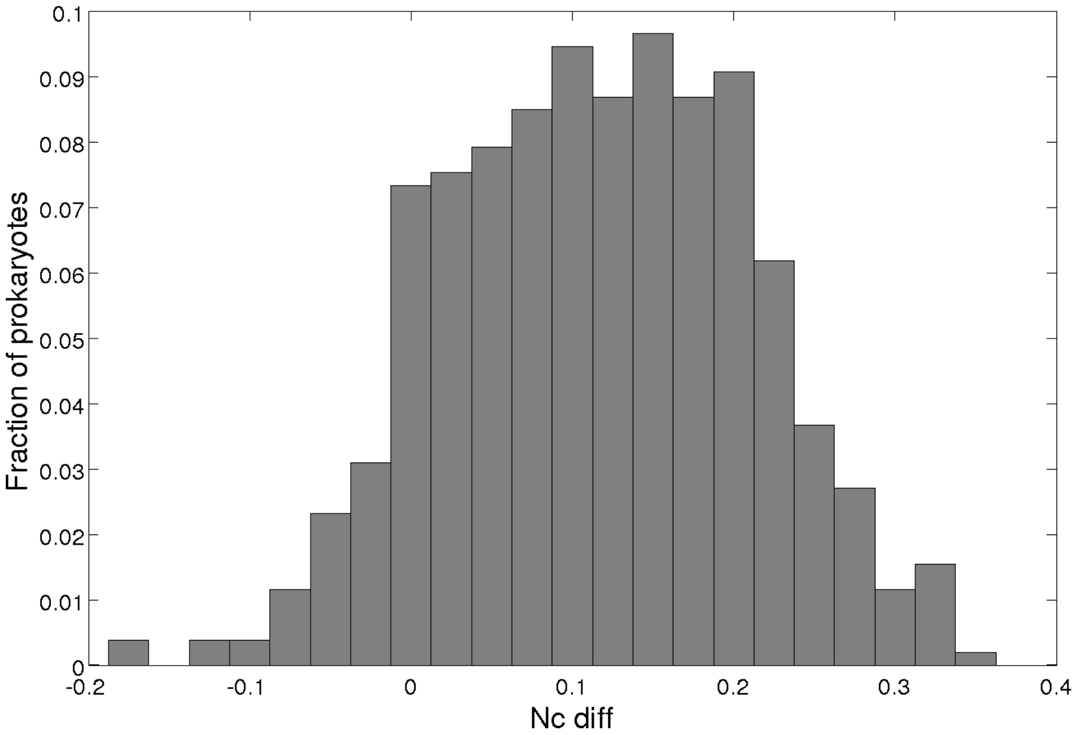

Figure S2. Distribution of Nc_{diff} values among 518 prokaryotes**.**

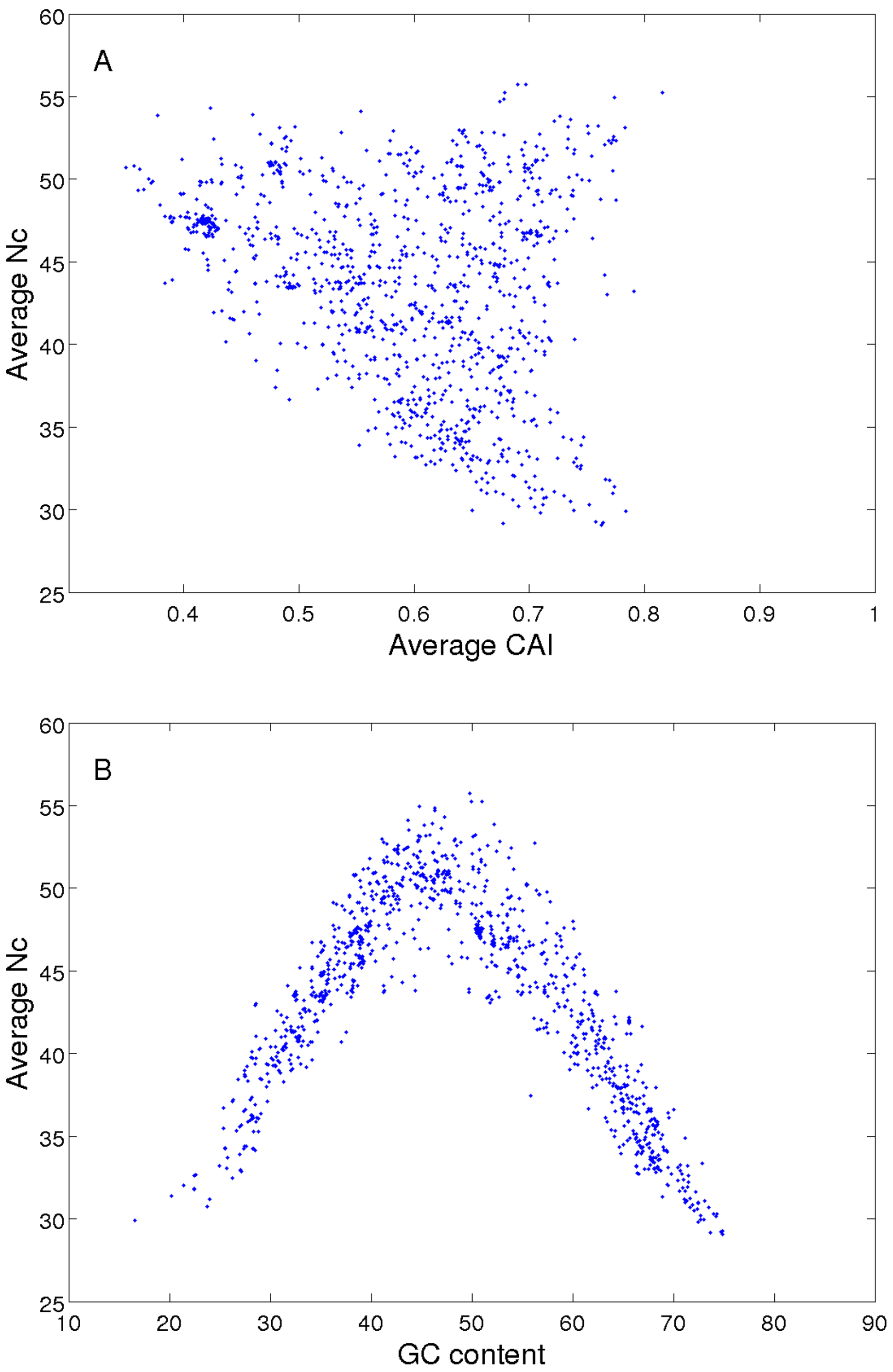

Figure S3. No correlation between Average Nc and CAI_{ave} values. [A] The average Nc value of a genome indicates if most genes in the genome use a limited set of codons (small value of average Nc) or many codons (high value of average Nc). As shown, unbiased genomes (with high CAI_{ave}) are of two types: genomes where all genes use a limited set of codons and genomes where there is a high number of codons used by all genes. CAI_{ave} is indicated as 'Average CAI'. **[B]** The values of average Nc strongly depend on the GC content of the genome.

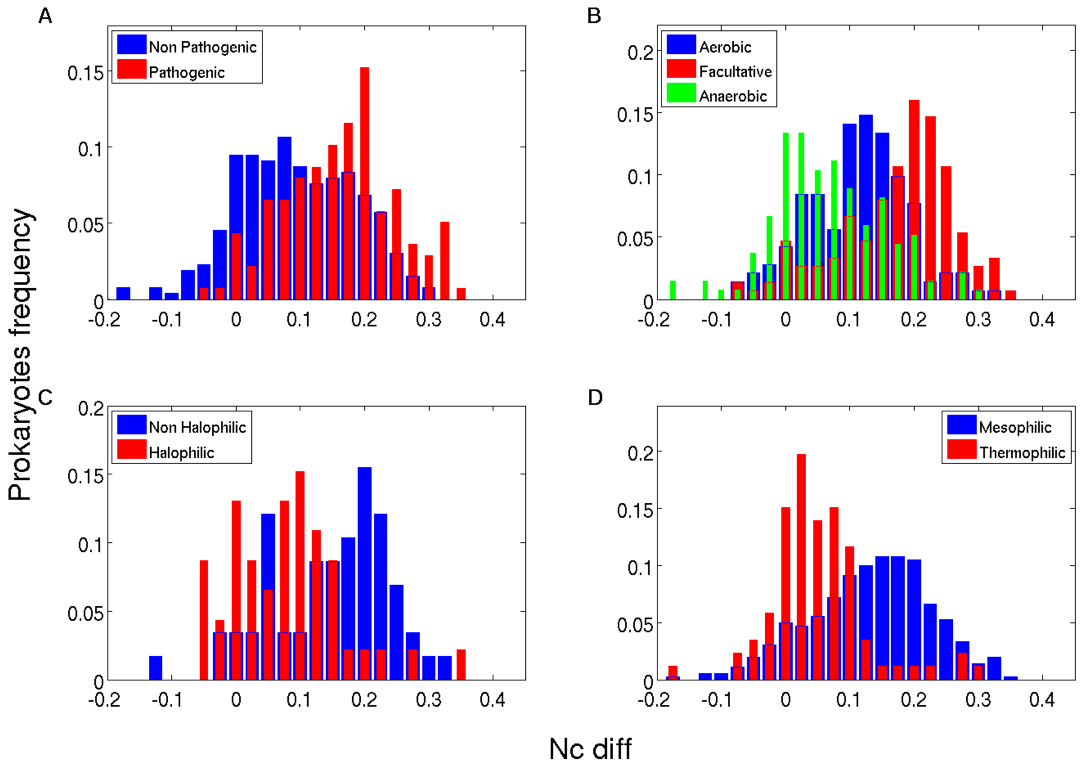

Figure S4. Prokaryotes exhibiting different environmental characteristics show different extents of codon usage bias (computed by Nc_{diff}). The data set of 518 prokaryotes was divided into groups according to various properties, and the distribution of Nc_{diff} values of the different groups were compared [A] Pathogenic prokaryotes show larger extents of codon usage bias than non-pathogenic prokaryotes (p=6.98E-11, Mann-Whitney test). [B] Facultative prokaryotes exhibit the highest extent of codon usage bias and anaerobic prokaryotes exhibit the lowest extent (p=5.87E-21, Kruskal-Wallis Test. p=1E-10, Mann-Whitney test between facultative and aerobic. p=8.39E-19, between facultative and anaerobic. p=7.16E-6, between aerobic and anaerobic). [C] Prokaryotes that live in different salinity environments show statistically significant differences in their Nc_{diff} values: halophilic prokaryotes are less biased than non halophilic (p=8.26E-5, Kruskal-Wallis test). [D] Thermophilic prokaryotes show a smaller extent of codon usage bias than mesophilic prokaryotes (p=9.88E-14, Mann-Whitney).

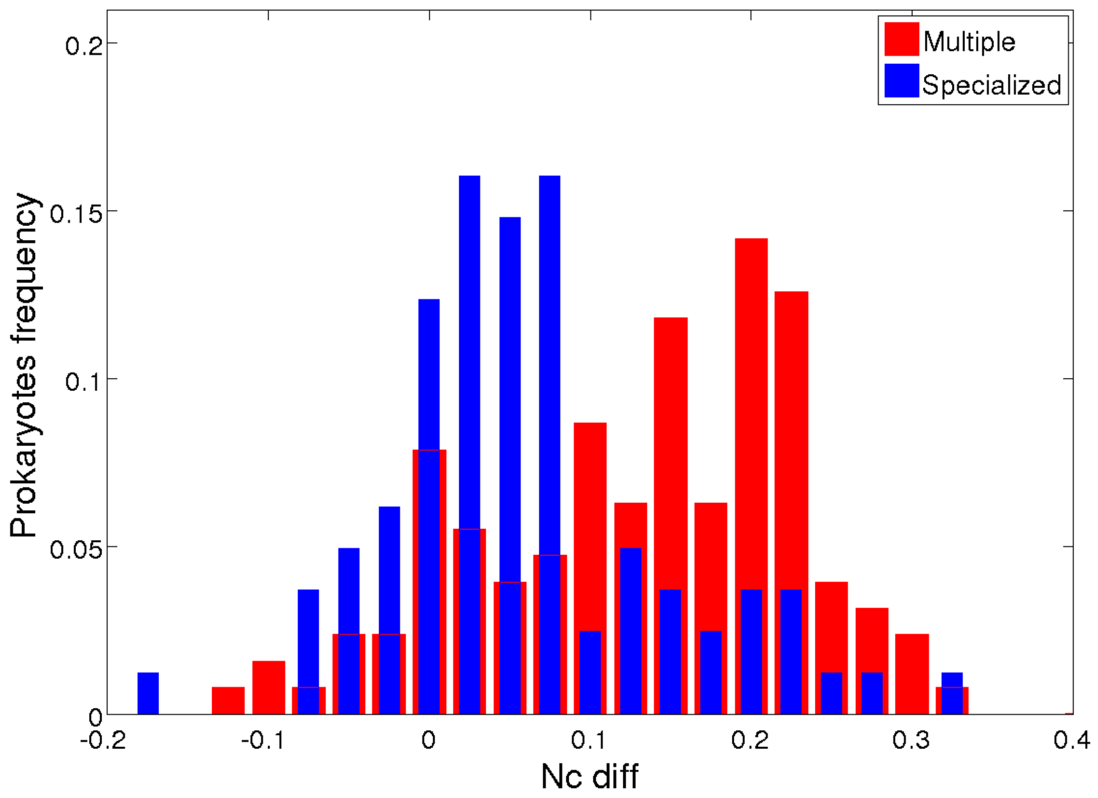

Figure S5. The ability of an organism to live in multiple habitats is the feature most correlated with the extent of codon usage bias. Prokaryotes living in specialized habitats demonstrated significantly lower Nc_{diff} values than organisms living in multiple habitats ($p=3.26E-7$, Mann-Whitney test).

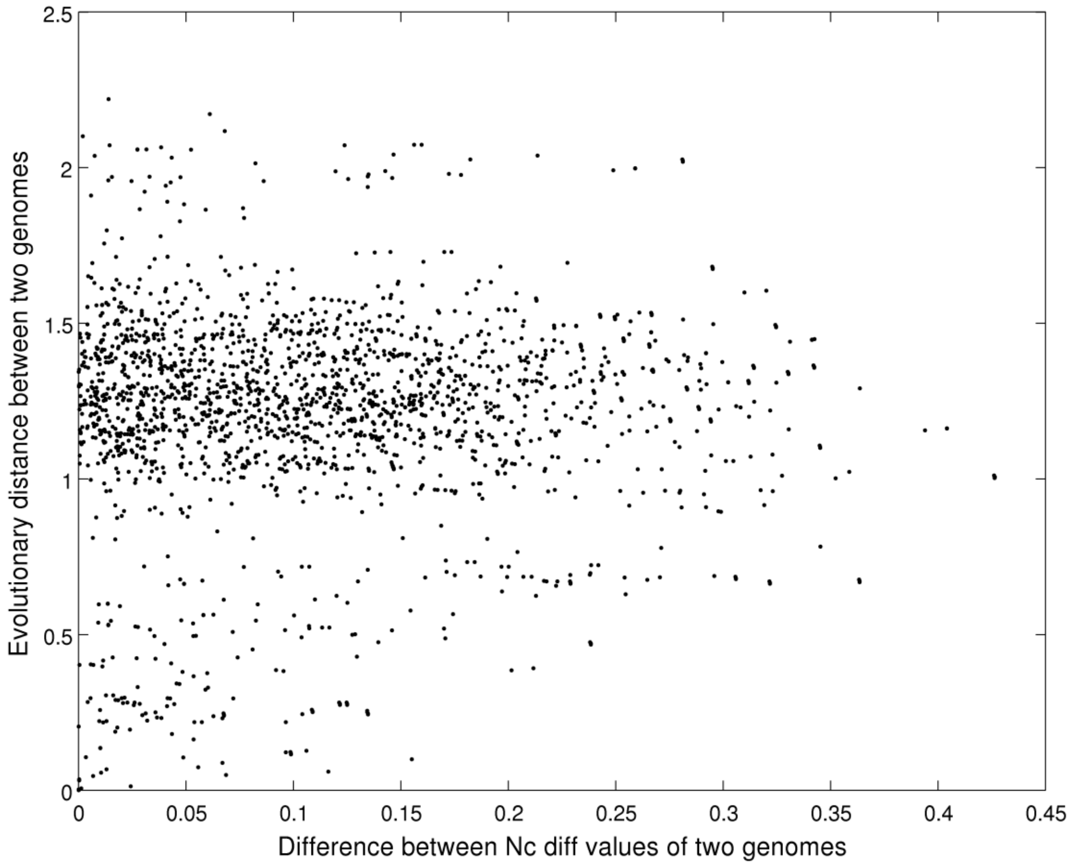

Figure S6. No correlation between phylogenetic distance and difference in Nc_{diff} values of pairs of organisms. The distance between pairs of bacteria or archaea was plotted against their Nc_{diff} values difference (Pearson r=0.04).

Table S1. Features and characteristics of the prokaryotes included in this study

A separate Excel file: Additional File 2.xls

Contains data and annotations of the organisms included in the analysis (name, tax ID, $CAI_{ave,}$ median CAI, coefficient of variation (of CAI), $Nc_{diff,}$ average Nc, environmental properties, if it the representative subspecies of the species, super kingdom, GC content).

Table S2. Statistical significance values of $\chi 2$ tests testing the interdependence between different phenotypic traits

	Pathogenicity	Oxygen requirement	Salinity	Temperature Range
Oxygen requirement	2.278E-7			
Salinity	2.945E-6	0.08875		
Temperature Range	1.838E-10	5.702E-9	0.008335	
Habitat	1.441E-6	6.675E-5	0.0001428	5.344E-26
